# Association between dietary patterns and prediabetes risk in a middle-aged Chinese population

**DOI:** 10.1186/s12937-020-00593-1

**Published:** 2020-07-30

**Authors:** Xiao-Ming Shen, Yi-Qian Huang, Xiao-Yan Zhang, Xiao-Qing Tong, Pei-Fen Zheng, Long Shu

**Affiliations:** 1 Department of Endocrinology, The No.1 People’s Hospital of Pinghu, Sangang Road Number 500, Danghu street, Pinghu, 314200 Zhejiang People’s Republic of China; 2 Department of Digestion, Zhejiang Hospital, Lingyin Road Number 12, Xihu District, Hangzhou, 310013 Zhejiang People’s Republic of China; 3 Department of Nutrition, Zhejiang Hospital, Lingyin Road Number 12, Xihu District, Hangzhou, 310013 Zhejiang People’s Republic of China

**Keywords:** Prediabetes, Dietary patterns, Factor analysis, Cross-sectional study, China

## Abstract

**Background:**

Information regarding dietary patterns associated with prediabetes in the Chinese population is lacking. The objective of the present study was to explore the association between major dietary patterns and the risk of prediabetes in a middle-aged Chinese population.

**Methods:**

A total of 1761 participants (aged 45 to 59 years) were recruited in Hangzhou city, the capital of Zhejiang Province, China from June 2015 to December 2016. Dietary information was obtained by interview using a 138-item, validated semi-quantitative food frequency questionnaire (SQFFQ). Multivariate logistic regression models were used to analyze the associations between dietary patterns and the risk of prediabetes with adjustment of potential confounding variables.

**Results:**

Three dietary patterns were ascertained by factor analysis and labeled as traditional southern Chinese, Western, and grains-vegetables patterns. After controlling of the potential confounders, participants in the top quartile of the Western pattern scores had greater odds ratio (OR) for prediabetes (OR = 1.54; 95% confidence interval (CI):1.068–2.059; *P* = 0.025) than did those in the bottom quartile. Compared with those in the bottom quartile, participants in the top quartile of the grains-vegetables pattern scores had a lower OR for prediabetes (OR = 0.83; 95% CI:0.747–0.965; *P* = 0.03). Besides, no statistically significant association was observed in the association between the traditional southern Chinese pattern and prediabetes risk (*P* > 0.05).

**Conclusions:**

The findings of this study showed that the Western pattern was associated with higher risk, and the grains-vegetables pattern was associated with lower risk of prediabetes. Future prospective studies are required to validate our findings.

## Introduction

Prediabetes is a condition of abnormal glucose homoeostasis such as impaired fasting glucose (IFG), impaired glucose tolerance (IGT) or a combination of both [1]. In China, the prevalence of diabetes has more than quadrupled in recent decades, with approximately 490 million adults in both urban and rural areas having prediabetes [2]. A previous study in 2014 reported that the prevalence of prediabetes was 28.7% based on fasting blood pressure (FBG) and 12.4% based on glycosylated Hb (HbA1c) in United States [3]. As far as we know, prediabetes is closely associated with numerous metabolic disorders, including obesity, hypertension and dyslipidaemia, which may contribute to increased morbidity and mortality [4, 5].^.^Evidence suggests that early intervention in the prediabetic population may help reduce the risk of type 2 diabetes mellitus(T2DM) by 40–70% [6]. Individuals with prediabetes may progress to normal plasma glucose levels after participating in lifestyle interventions.

In the past, a substantial amount of research has explored the influence of diet, a modifiable risk factor on diabetes risk, and the majority of these studies have examined individual foods or nutrients and their effect on diabetes [7, 8]. Nevertheless, in reality, individuals do not eat nutrients or foods in isolation, but consume meals containing combinations of many foods and nutrients that possibly interact with each other [9]. Consequently, dietary pattern analysis is now widely accepted in the realm of nutritional epidemiology as a more recognizable approach for assessing the relationship between diet and diseases, because it takes into account the complexity of whole-diet and potentially facilitates nutritional recommendations [10]. Up until now, considerable attentions have been focused in medical research on the association between overall dietary patterns and the risk of diabetes [11–19]. A recent meta-analysis by Jannasch et al., indicated that diets according to the Mediterranean diet, Dietary Approaches to Stop Hypertension (DASH), and Alternative Healthy Eating Index (AHEI) had a strong potential for preventing diabetes [20]. Besides, a recent systematic review and meta-analysis of observational studies also demonstrated that greater adherence to plant-based dietary patterns, especially when they are enriched with healthful plant-based foods, may be beneficial for the primary prevention of T2DM [21].

As many developing countries, China has also experienced a socioeconimic transition coupled with westernization in lifestyle and diet [22]. Accompanied by the nutrition transition, the burden of chronic diseases, e.g. diabetes, obesity are increasing in China [23]. Therefore, studying the relationship between dietary patterns and prediabetes can be of value. To date, many epidemiological studies have examined dietary patterns in relation to T2DM and gestational diabetes mellitus (GDM) in China [11–19]. Only two published studies, however, have been reported on the association of dietary patterns with prediabetes in Chinese population [5, 24]. Furthermore, to the best of our knowledge, no previous study has addressed the relationship between dietary patterns and risk of prediabetes in a middle-aged Chinese population. Therefore, this study was carried out to determine the associations between dietary patterns defined by posterior approach and the risk of prediabetes in Chinese adults aged 45–59 years.

## Subjects and methods

### Study population

This cross-sectional study was performed in the city of Hangzhou, the capital of Zhejiang Province in the people’s Republic of China from June 2015 to December 2016. The detailed description of this study has been reported previously [14]. A total of 2437 eligible participants were invited to attend the health checks at the Medical Center for Physical Examination, Zhejiang Hospital, where the participants were face-to-face interviewed by well-trained dietitians using written questionnaires. We excluded 453 participants with self-reported having diabetes,which may change their lifestyles and dietary habits. We additionally excluded 15 participants with a family history of diabetes. Further exclusion included 36 participants who provided incomplete anthropometric information, 138 participants who provided the missing information on their dietary intake, 14 participants who reported implausible energy intakes (< 600 or > 4000 kcal/d), 20 participants who didn’t provide blood sample. After exclusions, the final analysis was conducted on data from 1761 participants (914 male and 847 female). All participants provided written informed consent when recruited for the study.

### Dietary assessment

Dietary information was collected by well-trained dietitians, using a 138-item, validated semi- quantitative food frequency questionnaire (SQFFQ), which was designed to measure the dietary intake in middle-aged Chinese adults. The validity of reliability of SQFFQ has been previously validated [25]. Participants were requested to recall the frequency of 138 food items in the preceding year and the estimated portion size, using local weight units (1 Liang = 50 g) or natural units (cups). For each food item, the consumption frequency was reported using nine categories in this questionnaire, ranging from “never”, “< 1 time/month”, “1–3 times/month”, “1–2 times/week”, “3–4 times/week”, “5–6 times/week”, “1 time/day”, “2 times/day” to “3 times/day”. During face-to-face interviewing, the portion size of each food item was estimated using food models and standard serving sizes(e.g., one standardized portion of cooked rice is one small bowl, weighing approximately 100 g). Then, the frequency of intake and portion size were used to calculate the amount of each food item consumed on average, using the Chinese Food Composition Table as the database. Finally, these data were converted to grams or millilitres per day and used in the following analysis.

### Identification of dietary patterns

First, we collapsed 138 food items from the SQFFQ into 30 predefined food groups (Table S1) based on similarities in ingredients, nutrient profile, and culinary usage in a middle-aged Chinese population [14]. Before performing the factor analysis, the Kaiser-Meyer-Olkin Measure of Sample Adequacy and the Bartlett Test of Sphericity were used to assess data adequacy. If appropriate, factor analysis (principal component method) was used to derivate the major dietary patterns. During data analyses, the factors were rotated by orthogonal transformation (varimax rotation) to achieve simpler structure with greater interpretability; The eigenvalue and scree plot were applied to decide which factors to be remained [26]. After evaluating the eigenvalues, the scree plot test, and interpretability, factors with an eigenvalues ≥2.0 were retained. In the present study, the predefined food groups with a factor loading ≥|0.4| were considered as major contributors to a give dietary pattern. The labeling of dietary patterns was based on the interpretation of foods with high factor loadings for each dietary pattern [26]. Finally, dietary pattern scores were categorized according to quartiles with Q1 corresponding to the lowest quartile of dietary pattern score.

In our previous study [14], three dietary patterns have been extracted, naming the traditional southern Chinese pattern, which was characterized by high intakes of refined grains, vegetables, fruits, pickled vegetables, fish and shrimp, bacon and salted fish, salted and preserved eggs, milk, soya bean and its products, miscellaneous bean, fats, drinks; the Western pattern was characterized by high intakes of red meats, poultry and organs, processed and cooked meat, eggs, seafood, cheese, fast foods, snacks, chocolates, alcoholic beverages, coffee; the grains-vegetables pattern was characterized by high intakes of whole grains, tubers, vegetables, mushrooms,vegetable oil, nuts, honey, tea.

### Assessment of blood pressure

Blood pressure was measured twice by a trained nurse after a 5–10 min rest in the sitting position, using a standard mercury sphygmomanometer. The mean of two measurements was considered as the participant’s final blood pressure.

### Assessment of biomarker

All blood samples were drawn between 7:00 and 9:00 in the morning into evacuated tubes from each participant after 12 h of fasting overnight, and stored temporarily at − 20 °C until subsequent analyses. Then, to measure 2-h plasma glucose (2-h PG), participants underwent an oral glucose tolerance test using 75 g of glucose. All blood samples were analyzed in the Medical Center for Physical Examination, Zhejiang Hospital for fasting plasma glucose (FPG), 2 h PG, Glycosylated Hb (HbA1c), total cholesterol (TC), triglycerides (TG), high-density lipoprotein-cholesterol (HDL-C), low- density lipoprotein-cholesterol (LDL-C), serum uric acid (SUA), alanine aminotransferase (ALT) and asparagine aminotransferase (AST) using the Hitachi 7180 automatic biochemical analyzer (Hitachi, Tokyo, Japan).

### Assessment of anthropometric measurements

Weight in light clothes and without shoes was measured with an accuracy of 100 g with a digital scale, and height was measured using a stadiometer with an accuracy of 0.1 cm. Body mass index (BMI) was calculated using weight (kg) divided by the square of height(m^2^). Waist circumference (WC) was measured at the midpoint between the lower rib edge and the upper iliac crest by means of a metric measure with an accuracy of 1 mm [27]. All anthropometric measurements were conducted by well- trained nurse according to standard procedures.

### Assessment of other variables

Physical activity was assessed using the International Physical Activity Questionnaire (IPAQ), and expressed as metabolic equivalent hours per week (MET-h/week) [28]. Then, based on MET-h/week, three categories of physical activity were assigned, including light, moderate and heavy [29]. Additional information including smoking habits (never, current, and former smokers), education level (primary school or below, middle and high school, junior college or above) was recorded with a structured questionnaire. Moreover, total energy intake was estimated through this semi-quantitative FFQ, and results were expressed in kilocalorie per day (kcal/day) and categorized according to quartile.

### Definition of prediabetes

Prediabetes was defined as those without a previous diabetes diagnosis and the satisfaction of at least one of three conditions:(1) FPG test with values between 5.6 and 6.9 mmol/L;(2) HbA_1c_ of 5.7–6.4%;(3) the 2-h oral glucose tolerance test (OGTT) with values between 7.8 and 11.0 mmol/L [30].

### Statistical analyses

Data were analyzed across the quartiles of each dietary pattern score, and results for categorical variables were expressed as number (percentages), and continuous variables were expressed as mean ± standard deviation (SD). First, Kolmogorov-Smirnov test was used to test the normal distribution of the variables. The continuous variables with normal and non-normal distribution were compared using the Independent-Samples t and Mann-Whitney tests, respectively. In addition, we used the chi-squared test to assess the difference in categorical variables. Multivariate logistic regression analysis was performed to evaluate the relationship between dietary patterns and prediabetes, adjusting for potential confounders. Model 1 was adjusted for age (years) and sex (male/female); Model 2 was further adjusted for educational level(<high school, high school, >high school), smoking status (never, current, former), physical activity (MET-h per week), hypertension (yes/no) and BMI (continuous); Model 3 was additionally adjusted for total energy intake (kcal/d). All statistical analyses were conducted with the use of the IBM Statistical Package SPSS version 23.0(SPSS Inc., Chicago, IL, USA), and a 2-tailed *P* < 0.05 was considered statistically significant.

## Results

Of the 1761 eligible participants, 17.3% had prevalent prediabetes. The characteristics of study participants with and without prediabetes are shown in Table 1.There were significant differences for age, smoking status, education, income, and the prevalence of obesity between participants with and without prediabetes(*P* < 0.05). Participants with prediabetes were significantly younger (50.67 ± 7.92 vs 52.82 ± 8.06), smoker (27.4% vs 17.5%), higher educational level (18.9% vs 3.4%) and income (17.8% vs 11.1%), and higher prevalence of obesity (12.0% vs 6.3%) than those without prediabetes. Moreover, in our previous study [16], three major dietary patterns were identified, naming the traditional southern Chinese, western and grains-vegetables patterns, which explained 10.3, 8.5 and 6.8% of the dietary intake variance, respectively. The factor-loading matrixes for these dietary patterns are shown in Table S2.

**Table 1:** The characteristics of study participants with and without prediabetes

Variables	Participants with prediabetes (*n* = 305)	Participants without prediabetes(*n* = 1456)	Significance^*^
Demographic			
Age (years)	50.67 ± 4.52	52.82 ± 5.76	*P* < 0.001
Gender			
Male	173 (56.7)	741 (50.9)	χ^2^ = 3.432
Female	132 (43.3)	715 (49.1)	*P* = 0.064
Smoking status (%)			
Never	220 (72.1)	1153 (79.2)	χ^2^ = 9.060
Former	9 (3.0)	48 (3.3)	*P* < 0.05
Current	76 (24.9)	255 (17.5)	
Education (%)			χ^2^ = 113.3
Primary school or below	22 (7.2)	260 (17.9)	*P* < 0.001
Middle and high school	226 (74.1)	1145 (78.6)	
Junior college or above	57 (18.9)	51 (3.5)	
Monthly income per person(%)			
≤2500 (RMB)	31 (10.2)	652 (44.8)	χ^2^ = 127.3
2500–4000 (RMB)	219 (71.8)	642 (44.1)	*P* < 0.001
> 4000 (RMB)	55 (18.0)	162 (11.1)	
Obesity (%)			χ^2^ = 7.484
Yes	36 (11.8)	104 (7.1)	*P* < 0.01
No	269 (88.2)	1352 (92.9)	
Hypertension(%)			χ^2^ = 0.877
Yes	83 (27.2)	359 (24.7)	*P* = 0.349
No	222 (72.8)	1097 (75.3)	

Categorical variables are presented as sum and percentages, and continuous variables are presented as Mean ± SD

*Abbreviation*: *RMB* Ren min bi

^*^P values for continuous variables (Analysis of variance) and for Categorical variables (chi-square test)

The general characteristics of study participants across quartiles of the major dietary pattern scores are shown in Table 2.Compared with participants in the lowest quartile, those in the highest quartile of the traditional southern Chinese pattern were females, never-smokers and had higher level of 2-h glucose, higher prevalence of hypertension, lower total energy intake. In contrast, participants who belonged to the highest quartile of the western pattern were more likely to be younger, males, smokers, with higher BMI, WC, WHR, FBG, 2-h glucose, educational level, total energy intake and higher prevalence of obesity, hypertension, and prediabetes than those in the lowest quartile. Besides, in comparison with the participants from the lowest quartile of the grains-vegetables pattern, those in the highest quartile were more likely to be older, females, never-smokers, and had lower BMI, WC, WHR, FBG, 2-h glucose, economic income and total energy intake, and had lower prevalence of obesity, hypertension and prediabetes.

**Table 2:** The general characteristics of study participants across quartiles of the major dietary pattern scores

	Traditional southern Chinese pattern score		*P*	Western pattern score		*P*	Grains-vegetables pattern score		*P*
	Q1 (lowest) (*n* = 440)	Q4 (highest) (*n* = 441)		Q1 (lowest) (*n* = 440)	Q4 (highest) (*n* = 440)		Q1 (lowest) (*n* = 441)	Q4 (highest) (*n* = 440)	
Age (y)	51.6 ± 4.4	50.4 ± 4.3	0.252	51.7 ± 4.6	49.8 ± 4.3	**< 0.01**	50.3 ± 4.5	51.9 ± 4.7	**< 0.05**
BMI (kg/m^2^)	24.37 ± 2.96	24.99 ± 2.58	0.355	24.22 ± 2.67	25.24 ± 3.07	**< 0.05**	25.06 ± 2.57	24.13 ± 2.46	**< 0.05**
WC (cm)	84.55 ± 8.74	85.16 ± 8.12	0.411	84.30 ± 9.01	88.04 ± 8.62	**< 0.01**	87.10 ± 8.24	82.59 ± 8.80	**< 0.01**
WHR	0.87 ± 0.05	0.88 ± 0.06	0.564	0.87 ± 0.07	0.89 ± 0.06	**< 0.05**	0.88 ± 0.05	0.86 ± 0.08	**< 0.05**
FBG (mmol/L)	5.24 ± 0.63	5.35 ± 0.70	0.07	5.09 ± 0.80	5.81 ± 0.71	**< 0.01**	5.76 ± 0.84	5.37 ± 0.71	**< 0.01**
2-h glucose (mmol/L)	7.31 ± 0.91	7.08 ± 0.88	**< 0.05**	7.00 ± 0.98	7.72 ± 1.12	**< 0.001**	7.65 ± 1.03	7.29 ± 0.95	**< 0.01**
Obesity (%)	72 (16.4)	62 (14.1)	0.341	52 (11.8)	83 (18.9)	**< 0.01**	74 (16.8)	46 (10.5)	**< 0.01**
Hypertension (%)	127 (28.9)	159 (36.1)	**< 0.05**	104 (23.6)	144 (32.7)	**< 0.01**	135 (30.6)	108 (24.5)	**< 0.05**
Prediabete(%)	85 (19.3)	75 (17.0)	0.374	70 (15.9)	102 (23.2)	**< 0.01**	97 (22.0)	62 (14.1)	**< 0.01**
Gender (%)			**< 0.001**			**< 0.05**			**< 0.001**
Female	138 (31.4)	217 (49.2)		202 (45.9)	165 (37.5)		168 (38.1)	270 (61.4)	
Male	302 (68.6)	224 (50.8)		238 (54.1)	275 (62.5)		273 (61.9)	170 (38.6)	
Smoking status (%)			**< 0.001**			**< 0.01**			**< 0.01**
Never	362 (82.3)	322 (73.0)		325 (73.9)	300 (68.2)		340 (77.1)	380 (86.4)	
Current	65 (14.7)	111 (25.2)		72 (16.3)	113 (25.7)		73 (16.6)	38 (8.6)	
Former	13 (3.0)	8 (1.8)		43 (9.8)	27 (6.1)		28 (6.3)	22 (5.0)	
Education (%)			0.125			**< 0.01**			0.112
Primary school or below	151 (34.3)	176 (39.9)		136 (30.9)	164 (37.3)		170 (38.5)	162 (36.8)	
Middle and high school	257 (58.4)	243 (55.1)		277 (63.0)	230 (52.3)		228 (51.7)	250 (56.8)	
Junior college or above	32 (7.3)	22 (5.0)		27 (6.1)	46 (10.5)		43 (9.8)	28 (6.4)	
The average monthly income per person(%)			0.084			0.153			**< 0.01**
< 2000(RMB)	179 (40.7)	209 (47.4)		194 (44.1)	172 (49.1)		171 (38.8)	220 (50.0)	
2000–4000(RMB)	218 (49.5)	186 (42.2)		205 (46.6)	212 (48.2)		202 (45.8)	170 (38.6)	
> 4000(RMB)	43 (9.8)	46 (10.4)		41 (9.3)	56 (12.7)		68 (15.4)	50 (11.4)	
Physical activity (%)			0.879			0.203			**< 0.05**
Light	356 (80.9)	351 (79.6)		345 (78.4)	364 (82.7)		349 (79.1)	346 (78.6)	
Moderate	66 (15.0)	70 (15.9)		61 (13.9)	53 (12.1)		78 (17.7)	64 (14.6)	
Heavy	18 (4.1)	20 (4.5)		34 (7.7)	23 (5.2)		14 (3.2)	30 (6.8)	
Total energy (kcal/d)	2179.4 ± 148.5	1866.1 ± 156.3	**< 0.001**	1906.5 ± 162.6	2263.0 ± 185.2	**< 0.001**	2119.6 ± 157.4	1892.8 ± 195.1	**< 0.01**

Categorical variables are presented as sum and percentages, and continuous variables are presented as Mean ± SD

*Abbreviation*: *BMI* Body mass index, *WC* Waist circumference, *WHR* Waist hip rate, *FBG* Fasting blood glucose

* *P* values for continuous variables (Analysis of variance) and for Categorical variables (chi-square test)

The relationship between dietary patterns and prediabetes risk by multivariate logistic regression analysis is shown in Table 3. After controlling of sex, age, education, physical activity, smoking status, hypertension, BMI and total energy intake, participants in the top quartile of the Western pattern scores had greater OR for prediabetes (OR = 1.54; 95%CI:1.068–2.059; *P* = 0.025) than did those in the bottom quartile. Compared with those in the bottom quartile, participants in the top quartile of the grains- vegetables pattern scores had a lower OR for prediabetes (OR = 0.83; 95%CI:0.747–0.965; *P* = 0.030). In addition, no significant association was observed in the association between traditional southern Chinese pattern and prediabetes(*P* > 0.05).

**Table 3:** Multivariable adjusted ORs and 95% CIs for prediabetes across the quartile (Q) categories of dietary pattern scores in Zhejiang Province, China

	Traditional southern Chinese pattern Score			Western pattern Score			Grains-vegetables Pattern Score		
	Q1	Q4	***p***	Q1	Q4	***p***	Q1	Q4	***p***
Model 1	1.00	0.97 (0.950, 1.000)	0.028	1.00	2.14 (1.633, 4.240)	0.000	1.00	0.54 (0.382, 0.570)	0.000
Model 2	1.00	1.03 (0.837, 1.533)	0.095	1.00	1.72 (1.198, 2.683)	0.003	1.00	0.71 (0.506, 0.767)	0.002
Model 3	1.00	1.17 (0.757, 1.826)	0.151	1.00	1.54 (1.068, 2.059)	0.025	1.00	0.83 (0.747, 0.965)	0.030

*OR* Odds ratio, *95%CI* 95% confidence interval, *Model 1*adjusted for sex and age, *Model 2* further adjusted for educational level(<high school, high school, >high school), physical activityl (MET-h/week), smoking status (never, current, former), hypertension (yes/no) and BMI, *Model 3* additionally adjusted for total energy intake, *Q4* the highest quartile of dietary patterns, *Q1* the lowest quartile of dietary patterns (reference), *CI* confidence interval

## Discussion

Studies regarding the association of empirically derived dietary patterns and the risk of prediabetes in Chinese population are sparse. According to our knowledge, this is the first study to determine the relationship between major dietary patterns and prediabetes risk in a middle-aged Chinese population. The results show that the grains-vegetables pattern may decrease the risk, and the Western pattern may increase the risk of prediabetes. Besides, no significant association was observed in the association between traditional southern Chinese pattern and prediabetes.

In the current study, we found that the traditional southern Chinese pattern was not associated with decreased risk of prediabetes. Contrarily to our results, Erber et al. showed that foods high in fruits and vegetables appear to confer a higher risk for diabetes [31]. Although the traditional southern Chinese pattern shares some components with the “healthy” pattern, high salt and refined grains consumption as the well-established detrimental factors for diabetes [14, 32] may be important differences for this inconsistent result. Hence, the complex nature of this pattern may explain this null finding to some extent. First, fruits and vegetables contain high amounts of dietary fiber and antioxidants. In Chinese diabetic patients, Jiang and his colleagues have reported that increasing fiber intake may be an effective approach to reduce HbA1c level, improving glycemic control [33]. Lattimer et al., has also shown that higher consumption of dietary fiber can delay gastric emptying and decrease absorption of macronutrients, resulting in lower postprandial blood glucose and insulin level [34]. Besides, previous studies have also shown that antioxidant substances, e.g. vitamin C, vitamin E, and other carotenoids compounds abundant in fruits are significantly associated with lower risk of obesity, an important risk for T2DM [35]. On the other hand, higher carbohydrate, especially from refined grains(i.e. rice) intake is more common in Chinese population, which has been shown to increase the risk of obesity [36], a major risk factor for diabetes. Simultaneously, pickled vegetables that were loaded on this pattern contain high amounts of salt. Our previous study has shown that high intake of salt can increase the risk of hypertension [37], an important risk factor for diabetes. In short, the above possibilities can’t be ruled out in our study.

The Western pattern characterized by high consumption of red meats, processed and cooked meat, eggs, seafood, cheese, fast foods, snacks, chocolates, alcoholic beverages and coffee was significantly associated with an increase in the risk of prediabetes. Our findings are concordance with the results obtained from a previous systematic review and meta-analysis, which found that consuming higher amounts of red and processed meats, high-fat dairy and refined grains in the context of “unhealthy” dietary patterns was associated a 30% increased risk of diabetes [38]. This positive association may partly be attributable to western pattern’s unhealthy constituents, including red meat, processed and cooked meat and fast foods. First, processed and cooked meats contain a lot of salt and nitrite. As mentioned before, high salt intake can increase the risk of diabetes [37]. In addition, nitrate as one of the common added preservatives in processed meats can convert to nitrosamine compounds, which have been reported to increase the risk of diabetes [38]. Second, red meats are rich in saturated fat and cholesterol, which has been shown to be associated with an increased risk of prediabetes and glucose intolerance [39]. Third, high intakes of snack and chocolates with high glycaemic load could lead to an increase in weight gain, which can affect the metabolism of glucose and insulin sensitivity, thus increasing the risk of diabetes [40]. Finally, advanced glycation of high-fat products and meats can enhance oxidative stress and inflammatory factors that may be accompanied with insulin resistance and increase the possibility of developing diabetes [41].

In our analyses, we observed an inverse relationship between the grains-vegetables pattern and prediabetes. These findings are in line with a recent systematic review and meta-analysis that documented a significant inverse association between adherence to plant-based dietary pattern and the risk for developing T2DM [21]. Several potential mechanisms may explain the observed favorable relationship. First, whole grains and vegetables may bring a rich array of dietary fiber. Previous studies have found that high fiber intake was associated with decreased risk of diabetes [33, 34]. Second, whole grains, tubers and vegetables in this pattern have a low glycemic index, which have been documented to be associated with reduced risk of insulin resistance [42], a well-known risk factor for diabetes. Third, green tea is the most common drink in the Chinese population. High intake of green tea has been confirmed to have protective effects on the risk of diabetes [43].

## Strengths and limitations

This study holds several strengths. First, to the best of our knowledge, this is the first study to explore the association between dietary patterns and prediabetes risk among a middle-aged Chinese population. Our findings provided valuable information for the early prevention of prediabetes through the dietary modifications in a middle-aged Chinese population. Second, dietary information was obtained by face-to-face interview using a validated semi-quantitative FFQ, covering 138 food and beverage items. This FFQ enabled us to obtain more reliable information on dietary intake of participants in the preceding year. Besides, the validity and reproducibility of this FFQ has also been confirmed elsewhere [14]. Third, in multivariable logistic regression models, we have controlled for multiple potential confounders. Nonetheless, there are several limitations that need mentioning. First, it is a cross-sectional design study and we cannot determine the causal relationship between dietary patterns and prediabetes risk. Thus, the results are needed to be verified in future prospective study. Second, principal component analyses require several subjective decisions in determining the number of factors to be retained, the method of rotation of the initial factors and the naming of dietary patterns [10]. Third, the participants in this study were recruited in Hangzhou city and are not representative of the entire population of the people’s Republic of China. Therefore, our results may not be extrapolated to the general Chinese population.

## Conclusions

To conclusion, the results of this study showed that the western pattern was associated with an increased risk, and the grains-vegetables pattern was associated with a reduced risk of prediabetes in a middle-aged Chinese population. Our findings emphasize the importance of whole-diet in the early prevention of prediabetes. Further prospective studies are therefore required to confirm whether a true causal association exists.

## Supplementary information

**Additional file 1: Table S1.** Food grouping used in the dietary pattern analysis.

**Additional file 2: Table S2.** Factor-loading matrix for the three dietary patterns*.

## Data Availability

The datasets used and/or analyzed during the current study are available from the corresponding author on reasonable request.
