# Supplementary material for: Association between dietary patterns and prediabetes risk in a middle-aged Chinese population
Source: Nutr J. 2020 Jul 30;19:77. doi: 10.1186/s12937-020-00593-1 (PMC7393887; doi:10.1186/s12937-020-00593-1)
Supplement: Supplementary file 1 — Additional file 1: Table S1. Food grouping used in the dietary pattern analysis. [file 12937_2020_593_MOESM1_ESM.doc]

**Table S1.** Food grouping used in the dietary pattern analysis

| Food groups | Food items |
| --- | --- |
| Refined grains | Rice, porridge, rice in soup, noodles,instant noodles,steamed bun, wonton, dumplings,white breads, toasted bread |
| Whole grains | Corn, sorghum, millet, oats |
| Tubers | Sweet potato, potato, taro |
| Vegetables | Wild vegetables, green vegetable, spinach, green peppers, tomato Chinese cabbage, radish, cucumer, eggplant |
| Fruit | Apple,pears,peach, apricots, cherries, grapes, bananas, cantaloupe, watermelon, oranges, grapefruit, kiwi, strawberries and et al. |
| Pickled vegetables | Salted vegetables, Chinese sauerkraut |
| Mushrooms | Mushroom, shiitakes, enoki |
| Red meat | Pork, mutton, beef |
| Poultry and organs | Chicken, duck, liver, animal blood |
| Processed and cooked meat | Ham and sausage, sauced pork, roast duck |
| Fish and shrimp | Fish, shrimp |
| Eggs | Duck eggs, chicken eggs |
| Seafood | Sea fish, shrimp, crab,squid, jellyfis, shellfish |
| Bacon and salted fish | Salted meat and duck, salted fish |
| Salted and preserved eggs | Salted duck and chicken eggs, preserved eggs |
| milk | Liquid milk, milk powder, yoghurt |
| Cheese | Cheese |
| Soya bean and its products | Tofu, dried bean curd, soy milk |
| Miscellaneous bean | Mung beans, red beans, hemp beans |
| Fats | Lard, butter |
| Vegetable oil | Soybean oil, tea oil, rapeseed oil, olive oil |
| Fast foods | KFC, Mcdonald,fried dough sticks and twists, fried cakes,pizza |
| Nuts | Walnut, peanuts, almonds, melon seeds |
| Snacks | Cookies, sachima, bread, cake, ice cream, candy, sweets ,potato chips, shrimp roll, popcorn |
| Chocolates | Chocolates |
| Honey | Honey, hydromel |
| Drinks | Coca-cola, sprite, fruit and vegetable drink, fruits juice |
| Alcoholic beverages | Beer, fruit wine, grape wine |
| Tea | Tea,scented tea, wong Lo Kat |
| Coffee | Coffee |
