# Supplementary material for: Association between dietary patterns and prediabetes risk in a middle-aged Chinese population
Source: Nutr J. 2020 Jul 30;19:77. doi: 10.1186/s12937-020-00593-1 (PMC7393887; doi:10.1186/s12937-020-00593-1)
Supplement: Supplementary file 2 — Additional file 2: Table S2. Factor-loading matrix for the three dietary patterns*. [file 12937_2020_593_MOESM2_ESM.doc]

**Table S2**. Factor-loading matrix for the three dietary patterns*****

| Food groups | Dietary patterns | | |
| --- | --- | --- | --- |
|  | Traditional southern Chinese | Western | Grains-vegetables |
| Refined grains | 0.411 | - | - |
| Whole grains | - | - | 0.534 |
| Tubers | - | - | 0.471 |
| Vegetables | 0.407 | - | 0.638 |
| Fruit | 0.462 | - | - |
| Pickled vegetables | 0.509 | - | - |
| Mushrooms | - | - | 0.664 |
| Red meat | - | 0.563 | - |
| Poultry and organs | - | 0.502 | - |
| Processed and cooked meat | - | 0.520 | - |
| Fish and shrimp | 0.486 | - | - |
| Eggs | - | 0.346 | - |
| Seafood | - | 0.417 | - |
| Bacon and salted fish | 0.529 | - | - |
| Salted and preserved eggs | 0.414 | - | - |
| Milk | 0.360 | - |  |
| Cheese | - | 0.315 |  |
| Soya bean and its products | 0.400 | - | - |
| Miscellaneous bean | 0.414 | - | - |
| Fats | 0.447 | - |  |
| Vegetable oil |  | - | 0.392 |
| Fast foods | - | 0.407 | - |
| Nuts | - | - | 0.303 |
| Snacks | - | 0.517 | - |
| Chocolates | - | 0.435 | - |
| Honey | - | - | 0.595 |
| Drinks | 0.472 | - |  |
| Alcoholic beverages | - | 0.301 | - |
| Tea | - | - | 0.357 |
| Coffee | - | 0.390 | - |
| Variance of intake explained (%) | 10.3 | 8.5 | 6.8 |

*****Absolute values<0.4 were excluded for simplicity.
